# Role of mannitol metabolism in the pathogenicity of the necrotrophic fungus *Alternaria brassicicola*

**DOI:** 10.3389/fpls.2013.00131

**Published:** 2013-05-13

**Authors:** Benoit Calmes, Thomas Guillemette, Lény Teyssier, Benjamin Siegler, Sandrine Pigné, Anne Landreau, Béatrice Iacomi, Rémi Lemoine, Pascal Richomme, Philippe Simoneau

**Affiliations:** ^1^SFR 4207 QUASAV, UMR 1345 IRHS, Université d'AngersAngers Cedex, France; ^2^SFR 4207 QUASAV, INRA, UMR 1345 IRHSAngers Cedex, France; ^3^SFR 4207 QUASAV, Agrocampus-Ouest, UMR 1345 IRHSAngers Cedex, France; ^4^Plateforme d'Ingénierie et Analyses Moléculaires, Université d'AngersAngers Cedex, France; ^5^SONAS EA 921, SFR 4207, QUASAV UFR des Sciences Pharmaceutiques et d'Ingénierie de la Santé, Université d'AngersAngers Cedex, France; ^6^USAMVBucharest, Romania; ^7^Ecologie, Biologie des Interactions, UMR 7267 CNRS/Université de PoitiersPoitiers, France

**Keywords:** mannitol, *Alternaria brassicicola*, pathogenicity, null mutants, oxidative and drought stress, isothiocyanates, brassicicolin A

## Abstract

In this study, the physiological functions of fungal mannitol metabolism in the pathogenicity and protection against environmental stresses were investigated in the necrotrophic fungus *Alternaria brassicicola*. Mannitol metabolism was examined during infection of *Brassica oleracea* leaves by sequential HPLC quantification of the major soluble carbohydrates and expression analysis of genes encoding two proteins of mannitol metabolism, i.e., a mannitol dehydrogenase (*AbMdh*), and a mannitol-1-phosphate dehydrogenase (*AbMpd*). Knockout mutants deficient for *AbMdh or AbMpd* and a double mutant lacking both enzyme activities were constructed. Their capacity to cope with various oxidative and drought stresses and their pathogenic behavior were evaluated. Metabolic and gene expression profiling indicated an increase in mannitol production during plant infection. Depending on the mutants, distinct pathogenic processes, such as leaf and silique colonization, sporulation, survival on seeds, were impaired by comparison to the wild-type. This pathogenic alteration could be partly explained by the differential susceptibilities of mutants to oxidative and drought stresses. These results highlight the importance of mannitol metabolism with respect to the ability of *A. brassicicola* to efficiently accomplish key steps of its pathogenic life cycle.

## Introduction

Mannitol is a six-carbon non-cyclic sugar alcohol which appears to be widespread in the biosphere, with the noticeable exception of the animal kingdom. This polyol (i.e., alcohol containing multiple hydroxyl groups) is ubiquitous throughout the fungal kingdom and is considered as being the most abundant of all soluble carbohydrates within mycelia and fruit bodies (Lewis and Smith, 1967; Horer et al., 2001; Dulermo et al., 2009). In fungi, mannitol and its metabolism have been postulated to have a multitude of functions as either a carbohydrate reserve, in NADPH regeneration, in morphogenesis and conidiation or as a protection from environmental stress (Solomon et al., 2007). Furthermore, some studies have reported that mannitol has a role in pathogenicity of plant and animal pathogens (Chaturvedi et al., 1996a; Velez et al., 2008). Levels of mannitol were found to rise dramatically during plant infection by biotrophic or necrotrophic fungi and this accumulation was accompanied by increased expression of genes involved in the mannitol pathway (Voegele et al., 2005; Jobic et al., 2007; Dulermo et al., 2009).

Two hypotheses mainly emerged to explain the pathogenic significance of mannitol production by fungi. Firstly, fungal mannitol may be involved in the sequestration of carbohydrates from host. Since many plants are unable to metabolize mannitol, the conversion of plant hexoses into mannitol seems an ideal strategy for the fungal pathogen or mutualist, providing a means for fungi to store carbohydrates and reducing power in a form not accessible to the host (Ceccaroli et al., 2003; Dulermo et al., 2009). As *in planta* mannitol accumulation mainly occurred when conidiophores emerged, the latter authors suggest that this polyol could be necessary for spore survival or germination. Similar conclusions were suggested in the case of the biotrophic interaction between the rust fungus *Uromyces fabae* and its host plant *Vicia faba*, or during pathogenesis of *Sclerotinia sclerotiorum*, a *B. cinerea*-related necrotroph (Voegele et al., 2005; Jobic et al., 2007).

Secondly, mannitol is supposed to act as an antioxidant agent and protect fungal cells by quenching reactive oxygen species (ROS) produced by hosts in response to attack. Polyols can thus be powerful radical scavengers *in vitro* (Smirnoff and Cumbes, 1989; Voegele et al., 2005) and *in vivo* (Shen et al., 1997a,b). In the animal pathogen *Cryptococcus neoformans*, a mannitol low-producing mutant was hyper-susceptible to oxidative killing by normal human neutrophils and by cell-free oxidants, and was hypovirulent in mice (Chaturvedi et al., 1996a,b). Moreover, transgenic tobacco lines constitutively expressing a celery mannitol dehydrogenase (MDH) or a plasma membrane mannitol transporter were shown to have enhanced resistance to pathogenic *Alternaria* species (Jennings et al., 2002; Juchaux-Cachau et al., 2007). These results suggested that both plant-expressed proteins supported the metabolism of fungal secreted mannitol, thus rendering the pathogen more susceptible to reactive oxygen-mediated plant defense. This hypothesis was further strengthened by the fact that the constitutive expression of the MDH transgene did not affect the pathogenicity of the non-mannitol-secreting fungal pathogen *Cercospora nicotianae*.

**Figure 1:**
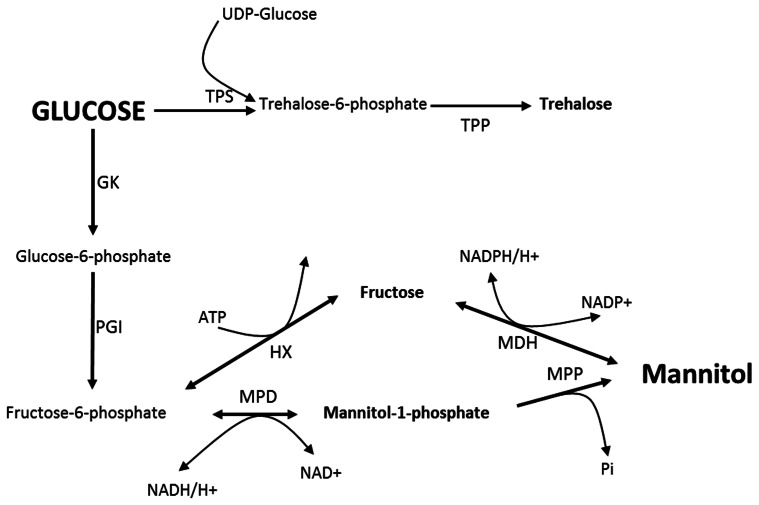
**Proposed mannitol cycle in fungi.** MDH, Mannitol dehydrogenase; MPP, Mannitol-1-P phosphatase; MPD, Mannitol-1-P dehydrogenase; HX, Hexokinase; PGI, Phosphoglucose isomerase; GK, Glucokinase; TPS, Trehalose-6-P synthase; TPP, Trehalose-6-P phosphatase.

Mannitol metabolism in fungi was initially thought to be a cyclical process (Hult and Gatenbeck, 1978). In this cycle (Figure 1), mannitol-1-phosphate 5-dehydrogenase (MPD; EC 1.1.1.17) was proposed to reduce fructose 6-phosphate into mannitol-1-phosphate using the NADH cofactor, followed by dephosphorylation by a mannitol-1-phosphate phosphatase (MPP; EC 3.1.3.22), resulting in inorganic phosphate and mannitol. Mannitol would then be oxidized to fructose by mannitol dehydrogenase (MDH; EC 1.1.1.138) using the NADP^+^ cofactor. Finally, fructose would be phosphorylated to fructose 6-phosphate by a hexokinase (HX; EC 2.7.1.1). Dephosphorylation of mannitol-1-phosphate into mannitol *via* MPP was described as being irreversible. Consequently, the proposed cycle would go in one direction with MPD as the major biosynthetic enzyme and MDH as a catabolic enzyme. However, recent data based on gene disruption experiments indicated that mannitol metabolism is not a cyclical process (Solomon et al., 2006; Velez et al., 2007; Dulermo et al., 2010). According to these reports, mannitol synthesis and degradation were both severely impacted by the loss of MPD, while the deletion of MDH appeared to have a more limited effect. Moreover, the *mdh* strains were found to be able to use mannitol as a sole carbon source, indicating that mannitol was not only catabolized by oxidation to fructose. Dulermo et al. (2010) recently reported the existence of a mannitol phosphorylation pathway in *B. cinerea*, suggesting that mannitol could be metabolized through mannitol-1-phosphate.

The behavior of *mpd* and *mdh* null strains *in planta* also questioned the importance of the mannitol pathway in fungal pathogenicity. Indeed, regardless of the fungus involved (*A. alternata*, *S. nodorum* or *B. cinerea*), the virulence of the *mpd* and *mdh* strains was not or partially compromised (Solomon et al., 2005, 2006; Velez et al., 2008; Dulermo et al., 2010). Nevertheless, mannitol was shown to be required for *in planta* sporulation, which is a crucial step in a polycyclic pathogen like *S. nodorum* (Solomon et al., 2005, 2006).

In this study, we investigated the role of the mannitol pathway in the plant necrotrophic fungus *Alternaria brassicicola*. This fungus causes black spot disease and is an economically important seed-borne fungal pathogen of Brassicaceae species. We isolated the genes encoding the MPD and MDH enzymes in *A. brassicicola* and used targeted gene disruption to create single and double mutants for each gene. We then explored the physiological functions of mannitol metabolism and, in particular, its involvement in *A. brassicicola* pathogenicity and in the protection of fungal cells against defense compounds [like isothiocyanates (ITC)] and other environmental stresses.

## Materials and methods

### Fungal strains and growth conditions

The *A. brassicicola* wild-type strain Abra43 used in this study has previously been described (Dongo et al., 2009; Joubert et al., 2011a). For routine culture, *A. brassicicola* was grown and maintained on potato dextrose agar (PDA) or on Vogel minimal medium (Vogel, 1956). For osmotic stress experiments mycelia were grown on PEG-infused agar plates (Verslues et al., 2006). Colony diameters were measured daily and used for calculation of radial growth. The method based on microscale liquid cultivation (from conidial suspensions) and automated nephelometric recording of growth, followed by extraction of relevant variables (lag time and growth rate), was described by Joubert et al. (2010). To study the susceptibility of fungal strains to ITC, allyl-ITC (AlITC), benzyl-ITC (BzITC) or phenetyl-ITC (PhITC), were diluted from stock solutions prepared in acetone at the final desired concentrations (2.5 and 5 mM). ITC were purchased from Aldrich Chemical Co. (Milwaukee, WI). To study the effects of plant extracts on mannitol accumulation, plant extracts were prepared from primary leaves of tomato or radish as described by Ehrenshaft and Upchurch (1993) and sterilized by filtration through a 0.2-μm nitrocellulose filter. Potato dextrose Broth (PDB) containing either 10% (v/v) aqueous plant-leaf extract or an equal volume of sterile distilled water were inoculated with conidia (10^5^ conidia/mL final concentration). Cultures were grown at 24°C with gentle agitation (150 rpm) for 7 days.

### Analysis of cell viability

Propidium iodide (PI) was used as a cell viability marker. Viable cells with intact membranes exclude PI, whereas the membranes of dead and damaged cells are permeable to PI. Fungal suspensions were prepared on PDB with conidia for 10^5^ conidia/mL (final concentration). Non-germinated conidia and germinated conidia after 15 h of incubation (150 rpm, 24°C) were treated with H_2_O_2_ (8 mM) or Al-ITC (5 mM). After 30 min of exposure, cells were washed twice with cold phosphate-buffered saline (137 mM NaCl, 2.7 mM KCl, 4.3 mM Na_2_HPO_4_.7H_2_O, and 1.4 mM KH_2_PO_4_, pH 7.4) and then stained with PI 2 μg/mL (Sigma-Aldrich).

### Generation of targeted gene replacement constructs and fungal transformation

The gene replacement cassettes were generated using the double-joint PCR procedure (Yu et al., 2004). The selectable marker inserted in the PCR constructs corresponded to the *Hph* gene cassette (1436 bp) from pCB1636 (Sweigard et al., 1995) or the *Nat* gene cassette (2150 bp) from pNR (Malonek et al., 2004) conferring resistance to hygromycin B and nourseotricin, respectively. The sets of primers used to amplify the 5′ and 3′ flanking regions of each targeted gene are presented in the Table 1. The double-joint final PCR products were used to transform *A. brassicicola* protoplasts as described by Cho et al. (2006). The *A. brassicicola* wild-type Abra43 was used to obtain single hygromycin resistant transformant strains Δ*abmpd* and Δ*abmdh*. The Δ*abmpd* genotype was used to obtain ΔΔ*abmpd-abmdh* hygromycin and nourseotricin resistant strains. Potential transformants were prescreened by PCR with relevant primer combinations (Table 1) to confirm integration of the replacement cassette at the targeted locus. Two putative gene replacement mutants for each construct were further purified by three rounds of single-spore isolation and then confirmed by Southern blot analysis.

**Table 1:** **List of primers for the genes used in this study**.

**Genes**	**Use**	**Primers**
*AbMdh*	Real-time PCR	F: TTGACACTGGCCTCTCCGAC
		R: GCCACAGCTTCTGGATGTCC
*AbMpd*	Real-time PCR	F: TTCCGAGCAAAACGGTTGAG
		R: CATTGTCCCACAGCAGCCT
*Hph*	Transformant validation	F: CGTTGCAAGACCTGCCTGAA
		R: GGATGCCGCTCGAAGTA
*Nat*	Transformant validation	F: TTCGGTTCCCTTTCTCCT
		R: ACATCCACGGGACTTGAGAC
*AbMdh*	5′ flanking regions for K.O.	F: GGCAAGTAAGTTGTGCGATTT
		R: TCCTGTGTGAAATTGTTATCCGCTGGAGGCACCAGTAACAATGA
*AbMdh*	3′ flanking regions for K.O.	F: GTCGTGACTGGGAAAACCCTGGCGCAATCACAGGGTTCCGATCT
		R: CCTCCTCCCATTCCAACATA
*AbMdh*	Nested for K.O.	F: GCGTTTCACGCGCTGGAGTATT
		R: GGGGCTGCGTTACAGAGGGAAGA
*AbMpd*	5′ flanking regions for K.O.	F: CGACCTTATCAGGCTTACGG
		R: TCCTGTGTGAAATTGTTATCCGCTAGGTCAATGGCATCGAAAAG
*AbMpd*	3′ flanking regions for K.O.	F: GTCGTGACTGGGAAAACCCTGGCGGTGCGTGTGTGTGTGTGTGT
		R: TAATGTGTTGGGAGGTGCAA
*AbMpd*	Nested for K.O.	F: TGGGTCTTCTTTGCTGTGTG
		R: GGGAAGACGTTGGGCAATCACT
*Hph* or *Nat*	Complementary tail for pcb1636 or pnr2	F: GTCGTGACTGGGAAAACCCTGGCG
		R: TCCTGTGTGAAATTGTTATCCGCT
*AbMpd*	5′ flanking regions for fusion GFP	F: ACATATATACCCCGCCAACG
		R: CTCCTCGCCCTTGCTCACCATGCCGCCGCCGCTGCTCTCCTGGACCTT
*AbMpd*	3′ flanking regions for fusion GFP	F: TCCTGTGTGAAATTGTTATCCGCTATATCCGCCAGTAAACTCTGAG
		R: AAGCGGATTGGGTCTTCTTT
*AbMpd*	Nested for fusion GFP	F: TTCTCACCCACTCCTCCAAC
		R: AACGGCTTGAAATGGACAAC
*AbMdh*	5′ flanking regions for fusion GFP	F: CTCCACATCAGCCTCCATCT
		R: CTCCTCGCCCTTGCTCACCATGCCGCCGCCCCTGACGCAGTAGCCACCGT
*AbMdh*	3′ flanking regions for fusion GFP	F: TCCTGTGTGAAATTGTTATCCGCTACGTATCGTTCCGCAAGGCC
		R: CACGCATCGCGTAGTTTTT
*AbMdh*	Nested for fusion GFP	F: CCCAAACTTCTCTACTCCCTCA
		R: GTACCACGACGGTTCACTCC
actin	Real-time PCR	F: GGCAACATTGTCATGTCTGG
		R: GAGCGAAGCAAGAATGGAAC
Gfp and *Hph*	Complementary tail for pCT74	F: CTCCTCGCCCTTGCTCACCAT
		R: TCCTGTGTGAAATTGTTATCCGCT

F, forward primer; R, reverse primer.

### Generation of fusion protein constructs

**Figure 2:**
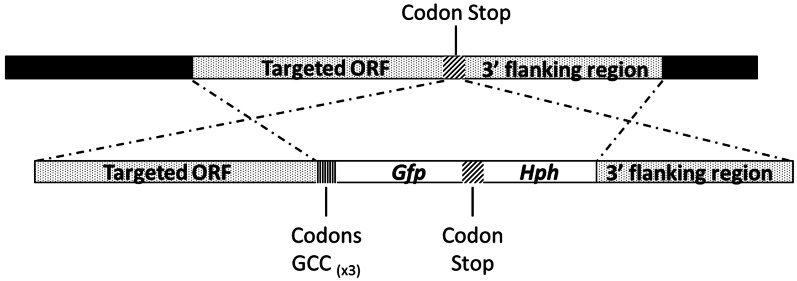
**Schematic representation of the *AbMpd* and *AbMdh* loci (dotted boxes) and replacement constructs with the GFP and HygB resistance (*Hph*) genes (white boxes)**.

The *Abmdh* or *Abmpd* C-terminal GFP fusion constructs were generated by fusion PCR (Figure 2). Using *A. brassicicola* genomic DNA as a template, the respective ORFs and 3′ flanking regions were amplified with relevant primer combinations (Table 1). In parallel, a fragment containing the *GFP* cassettes and *Hyg B* cassettes were amplified from the plasmid pCT74 (Lorang et al., 2001). The resulting PCR fragments were mixed and subjected to second fusion PCR. A linker containing 3 glycine residues was introduced at the 3′ end of the respective ORFs to replace the stop codons. The final PCR products were transformed in the *A. brassicicola* wild-type to make AbMpd- and AbMdh-GFP fusion proteins. The transformants with expected genetic integration events were identified by PCR and Southern blot analyses (data not shown).

### Nucleic acid isolation and analysis

Genomic DNA extraction and Southern blot analysis were conducted as previously described by Joubert et al. (2011a). Total RNA extractions and amplification experiments were conducted as previously described (Joubert et al., 2011b) using specific primers for *AbMdh* and *AbMpd* genes (Table 1).

### Infection assays

For plant infection assays on *Brassica oleracea* plants (var. Bartolo), 5 μL drops of *A. brassicicola* conidia suspension (10^5^, 10^4^ or 10^3^ conidia/mL in water) were inoculated on leaves from 5 weeks-old plants. Inocula were symmetrically deposited on the left and right sides from the central vein. The plants were then maintained under saturating humidity (100% relative humidity). Symptoms were observed and samples collected at 2, 4, 6 days post-inoculation (dpi) for the determination of major soluble carbohydrates contents and *AbMpd* and *AbMdh* expression analysis. For *in planta* sporulation assays, symptomatic tissues were sampled and vortexed for 30 s in water containing Tween 20 (0.02%, v/v). The concentration of the resulting conidia suspensions was estimated microscopically using a haemocytometer. For the microscopic analyses, *B. oleracea* leaf fragments were discolored, cleared and fungal structures were stained with solophenyl flavine 7GFE 500 (Ciba Specialty Chemicals, North Carolina, USA) as described by Hoch et al. (2005). Specimens were observed under a Leica fluorescent microscope (using 480 nm excitation and 527 nm emission).

### Seed contamination assessment

Seed contamination assessments were estimated as described by Pochon et al. (2012). Two 2.5 μL drops of an *A. brassicicola* conidial suspension (1 × 10^5^ conidia mL^−1^ in water) supplemented with 0.01% (v/v) Tween 20 were placed on the five youngest siliques (one drop at the silique base and one in the middle) from 1-month-old *A. thaliana* (L*er*) plants. At least five plants per fungal genotype were inoculated and the experiment was repeated twice. As a control for all experiments, two 2.5 μL drops of a 0.01% (v/v) Tween 20 solution were placed on five siliques of one plant. The plants were then maintained under saturating humidity for 2 days in the dark. Contaminated siliques were harvested 10 dpi. Inoculated or control siliques were dissected with sterile forceps and seeds were carefully harvested to avoid contact with the fungus potentially present on the outer surface of siliques. Seeds were incubated separately on PDA medium for 2 days. A seed was considered as contaminated when incubation resulted in typical *A. brassicicola* colony development.

### Conservation of conidia on seeds

*B. oleracea* seeds were artificially inoculated by incubation (1 h) in a conidia suspension (5 mL at 10^5^ conidia/mL). After removing the solution, the seeds were air-dried for 2 h and separated into two batches. The initial contamination rate was determined on one seed batch before storage. One seed per microplate well was placed in 300 μl of PDB and fungal growth was recorded in a laser-based nephelometer. The mean lag time was calculated and representative of the initial seed infection rate. The second seed batch was stored in a dry dark place at 24°C for 6 months and processed as above. As the lag time was found to be directly proportional to the number of germinating conidia, the viability rate was estimated from the ratio between lag times before and after storage. This experiment was repeated twice for each fungal genotype.

### Enzyme assays

Three-day-old cultures grown in PDB medium were harvested, submerged in liquid nitrogen for 5 min and stored at −80°C until use. Extraction procedures for cell-free extracts were carried out at 4°C and MPD and MDH enzymatic activities were measured as described by Velez et al. (2007). For both enzymes, specific activities were defined as the μmole of NADP(H) or NAD(H) oxidized per minute per mg of protein. Three independent experiments were done for each sample.

### Sugar and sugar alcohol extraction and analysis

Ethanolic extractions of cells from each sample used for hexose sugar and polyol measurements were performed as described by Stoop and Pharr (1993) with minor modifications. Dry powdered samples were suspended in 80% ethanol solution and incubated at 80°C for 5 min. After 5 min of centrifugation at 1000 g, the supernatants were recovered and pellets were re-extracted twice. Pooled ethanolic solutions were evaporated using a vacuum concentrator (speedVac UNIEQUIP), and residues were dissolved in sterile water or D_2_O for analysis. High performance liquid chromatography (HPLC) was performed on a Carbopac PA-1 column (Dionex Corp., Sunnyvale, CA, USA) as described by Rosnoblet et al. (2007). For each sample, three independent experiments were done from separate cultures.

### Detection of brassicicolin A from fungal extracts

Brassicicolin A was extracted as described by (Gloer et al., 1988) from cultures (minimal medium plus thiamine, Pedras et al., 1997) of Abra43 and ΔΔ*abmpd-abmdh* strains. Each filtered culture broth (1 L) was extracted with ethyl acetate (3 × 300 ml), and the organic phase was dried over MgSO_4_ and evaporated to generate 25 mg and 13.5 mg of crude extracts from Abra43 and ΔΔ*abmpd-abmdh* strains, respectively. Liquid chromatography-mass spectrometry (LC/MS) was performed on each extract using a Bruker Esquire 3000 Plus electrospray ionization-ion trap mass spectrometer coupled with a Waters 2790 high performance liquid chromatography (HPLC-ESI-MS^n^). Elution was carried out on an Hypersil RP18 column (250 × 4.6 mm, 5 μm, Termo) using the following gradient: initial mobile phase AcN/H_2_O 0.01% formic acid 15/85 reaching 60/40 in 35 min and maintained for 10 min before reaching 100/0 (v/v) until 46 min, with a flow rate of 1 mL/min. Only HPLC grade solvents were used. All samples were diluted in a solution of acetonitrile and filtered (UptiDisc™ PVDF 0.22 μm) prior to HPLC injection. They were analysed at 10 mg/ml concentration. The ESI parameters were as follows: solvent split ratio 1:9; nebulizer: 30 psi; dry gas (N_2_): 7 L/min; dry temperature: 340°C; skim: 40 V; trap drive: from 90 to 178, octopole RF amplitude: from 144 to 210 Vpp; capillary exit: from −156 to −240, capillary voltage 4500 V. The ion trap mass spectrometer was run in negative ion scanning mode for *m/z* ranging from 80 to 1500. MS^n^ was performed at a fragmentation amplitude ranging from 0.8 to 2.0 V depending on the samples. Preparative thin-layer chromatography (PTLC) was carried out on silica gel 60F254 (0.25 mm, Merck) using MeoH/CHCL_3_ (5/95) as eluent. This experiment was done twice from separate cultures of each fungal genotype.

### NMR analysis

For nuclear magnetic resonance (NMR) metabolite analysis, 500 μl of the D_2_O-samples were transferred to a 5 mm NMR tube, then analyzed on a BRUKER Advance DRX 500 MHz spectrometer equipped with a multinuclear QNP probe (Bruker, Wissembourg, France). Proton-decoupled ^13^C NMR spectra (sweep width = 31 450 Hz) were recorded at 125 MHz excitation frequency, 30-degrees pulse angle (6.5 μs pulse duration) at 2 s intervals. The free induction decays were collected as 32 K data points and processed with a 1–2 Hz exponential line broadening for ^13^C NMR. Maleic acid (δ_CH_ 130.4 ppm) was the external reference for chemical shifts. Identifications were made by comparison with spectra of pure known standards. For brassicicolin A, ^1^H (500 MHz) and 2D NMR spectra (HMQC and COSY) were recorded in CDCl_3_ in a capillary probe (Bruker TXI 1.7 mm) with chloroform resonances (δ_H_ 7.28, δ_C_ 77.0 ppm) as internal references. For each sample, NMR analysis was done twice.

## Results

### Characterization of Mpd and Mdh genes in *A. brassicicola* and generation of replacement mutants

**Figure 3:**
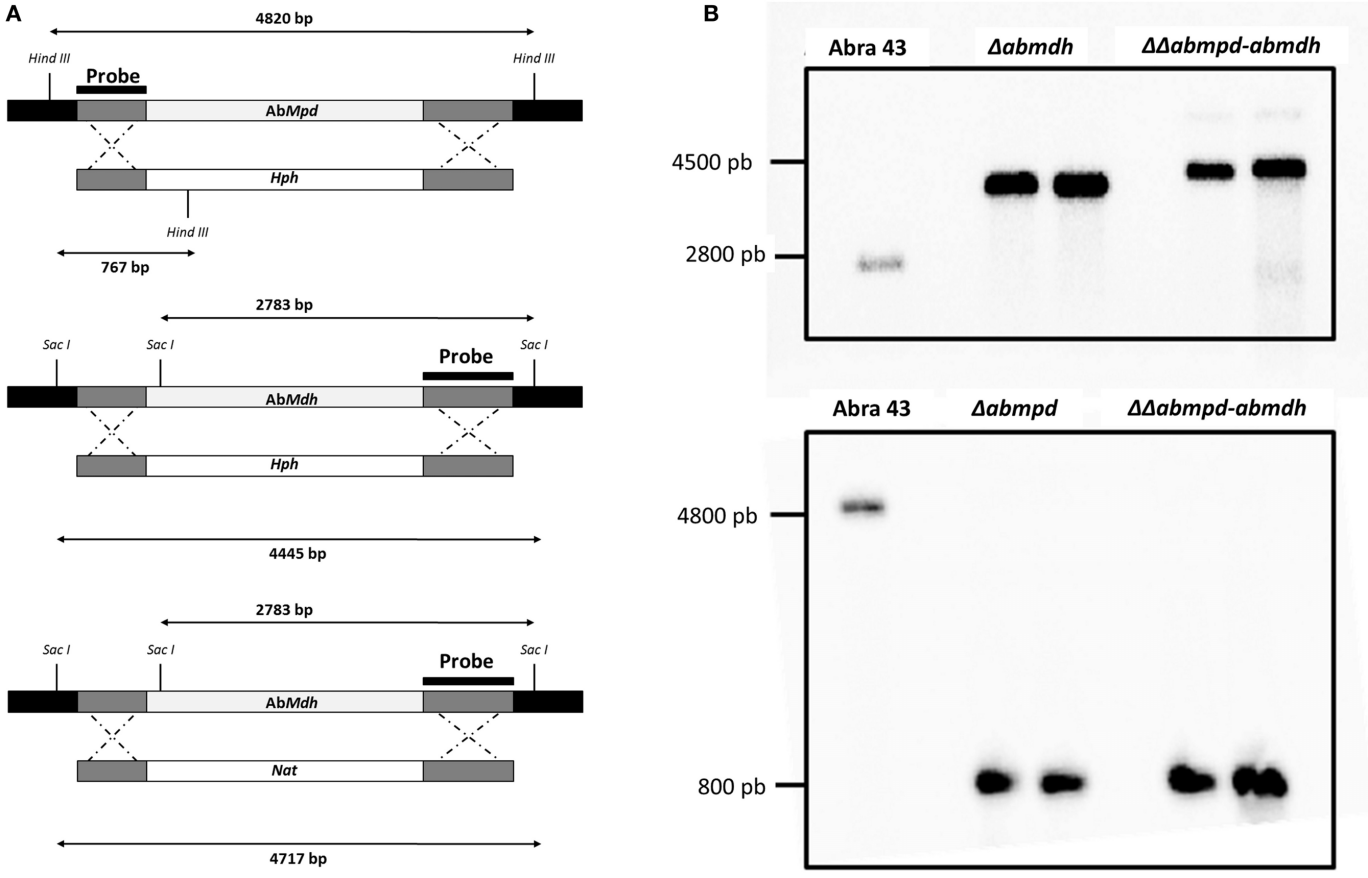
**Verification of deletion mutants. (A)** Schematic representation of the *AbMpd* and *AbMdh* loci (gray boxes), replacement constructs with the HygB resistance gene (*Hph*) or the nourseotricin resistance gene (*Nat*) cassettes (white boxes) and flanking sequences (dark gray boxes). The positions of *Hin*dIII and *Sac*I sites are indicated. **(B)** Southern hybridization of genomic DNA from wild-type Abra43 and transformants (for each targeted gene, two replacement mutants were generated and analyzed). Each DNA was digested with *Sac*I for the blot hybridized with the *Abmdh* probe or with *Hin*dIII for the blot hybridized with the *Abmpd* probe. Probes used are shown in **(A)**.

The presumed *Mpd* and *Mdh* loci were identified by a homology search against the *A. brassicicola* genome assembly (http://genome.jgi-psf.org/Altbr1/Altbr1.home.html) with genes previously described in *A. alternata* (Velez et al., 2007). *AbMdh* and *AbMpd* sequences (GenBank accession No JX403801 and JX403800, respectively) consisted of 851 and 1173 nucleotides, respectively. Blast search on the whole genome sequence and Southern analyses suggested the presence of only one copy of each gene (Figure 3B). Among the putative regulatory elements identified on sequences upstream of the ATG, consensus sequences for the binding of transcription factors involved in response to thermal, osmotic and oxidative stresses (Msn2p/Msn4p, HsF2) were found on the two genes.

The resulting AbMdh protein belongs to the short-chain group of the dehydrogenase/reductase superfamily (Jornvall et al., 1995) and has 95, 88, and 75% identity to the corresponding proteins described in *A. alternata*, *S. nodorum*, and *B. cinerea*, respectively. The AbMpd amino acid sequence shares 92, 82, and 57% identity with that of *A. alternata*, *S. nodorum*, and *B. cinerea*, respectively, and contains both NAD-interacting domains and a specific mannitol-1-phosphate dehydrogenase motif.

For each targeted gene, 2 replacement mutants (called Δ*abmdh1-2*, Δ*abmpd1-2*) were generated by replacing the targeted ORF with a hygromycin B resistance cassette. Two ΔΔ*abmpd-abmdh1-2* double deletion mutants were then constructed by transforming the Δ*abmpd* genotype with an *AbMdh*-replacement cassette containing a nourseothricin-resistance marker (Figure 3). In all further experiments, the phenotypic characters for transformants of the same genotype were not found to be significantly different.

### Biochemical characterization of replacement mutants

To confirm that gene inactivation impacted the enzyme activity, the transformants were grown in liquid culture and analysed for their ability to reduce fructose using NADPH as cofactor, or for fructose-6-phosphate conversion to mannitol-1-phosphate. Enzymatic assays (Table 2) confirmed that *AbMdh* and *AbMpd* deletions abolished mannitol dehydrogenase and mannitol-1-phosphate dehydrogenase activities, respectively.

**Table 2:** **Enzyme activities related to mannitol metabolism in mycelia of Abra43 wild-type and mutant strains**.

**Strains**	**MDH activity μmol min^−1^ mg of protein^−1^**	**MPD activity μmol min^−1^ mg of protein^−1^**
Abra43	3.7 ± 0.16	5.1 ± 0.85
Δ*abmpd*	3.7 ± 0.03	0
Δ*abmdh*	0	5.6 ± 0.35
ΔΔ*abmpd-abmdh*	0	0

MDH activity was measured in extracts as the rate of mannitol-dependent conversion of NAD^+^ to NADH and MPD activity was measured in extracts as the rate of mannitol-dependent conversion of NADP^+^ to NADPH (U). Data represent the means of three independent experiments.

**Figure 4:**
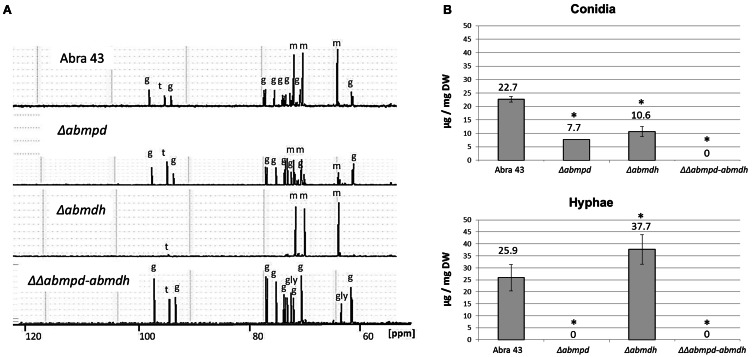
**Sugar and polyol content during *in vitro* development of *A. brassicicola* wild-type and mutant strains. (A)** ^13^C-NMR spectra obtained from ethanolic extracts of 1-week-old fungal colonies. The peaks were identified and labeled as follows: g, glucose; gly, glycerol; m, mannitol; t, trehalose. The spectra scales are identical and the chemical shift was expressed in parts per million (ppm). This experiment was done twice and representative spectra are presented here. **(B)** Mannitol content [expressed in μg/mg DF (dry weight)] obtained by HPLC analysis of extracts from mature conidia and young non-sporulating mycelium. Mannitol was extracted from at least three independent biological replicates. Error bars indicate standard deviations. Asterisks indicate a significant difference between the mutant and the parental isolate (Student test, *P* < 0.01).

The effects of the Δ*abmdh*, Δ*abmpd*, and ΔΔ*abmpd-abmdh* mutations on the accumulation of sugars and sugar alcohols were estimated by ^13^C NMR. Ethanolic extracts of sporulating mycelium grown in synthetic Vogel medium with glucose (2%) were obtained and analysed (Figure 4A). The wild-type and Δ*abmpd* extracts exhibited similar sugar profiles. However, lower amounts of mannitol and higher amounts of trehalose were found in the latter genotype. Conversely, the ^13^C-NMR spectra of Δ*abmdh* mutants were dominated by mannitol resonances. Mannitol was absent in extracts from ΔΔ*abmpd-abmdh* mutants in which trehalose and glycerol appeared to be the major compounds. Quantitative estimations of the mannitol content in the different genotypes during *in vitro* development were obtained by HPLC analysis of extracts from mature conidia and young non-sporulating mycelia (Figure 4B). While the wild-type accumulated nearly the same amount of mannitol in conidia and mycelia, this polyol was exclusively detected in conidia of the Δ*abmpd* mutants. By contrast, the Δ*abmdh* mutants preferentially accumulated mannitol in mycelia. No mannitol was detected in either conidia or young mycelia (30 h post germination) from the ΔΔ*abmpd-abmdh* mutants. However, traces of mannitol were detected from the double deletion strains in 1-week-old cultures (data not shown).

### Susceptibility of replacement mutants to stress conditions

**Figure 5:**
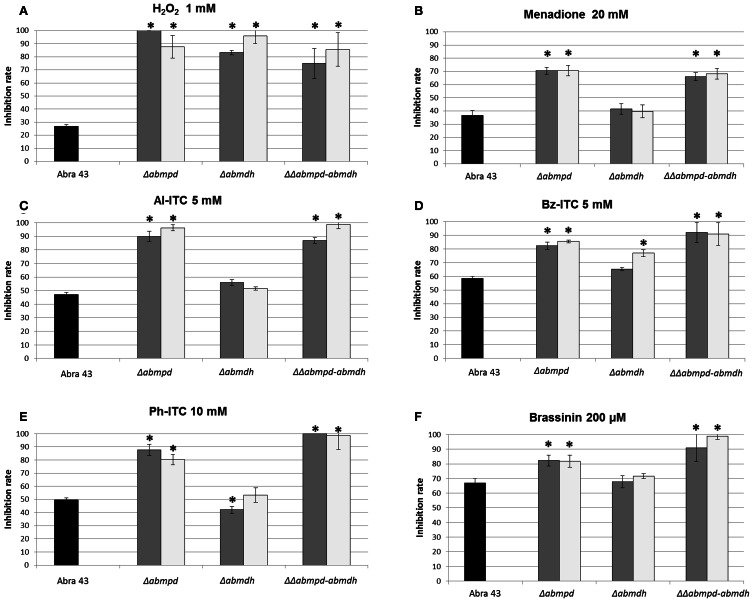
**Growth inhibition rates of the wild-type strain (black bars) and mannitol metabolism deficient mutants (2 transformants by genotype, gray, and white bars) in the presence of several molecules: H_2_O_2_ (1 mM) (A), menadione (20 mM) (B), Al-ITC (5 mM) (C), Bz-ITC (5 mM) (D), Ph-ITC 10 mM (E) and brassinin (200 μM) (F).** The results are expressed as the percentage of inhibition in treated samples compared to the control without additive. Conidia were used to inoculate microplate wells containing standard PDB medium that was supplemented with the appropriate test substance. Growth was automatically recorded for 25 h at 25°C using a nephelometric reader (see Materials and Methods/Fungal strains and growth conditions). Each genotype was analyzed in triplicate and the experiments were repeated three times per growth condition. Error bars indicate standard deviations. Asterisks indicate a significant difference between the mutant and the parental isolate (Student test, *P* < 0.01).

Mannitol has been proposed to act as a potent protective metabolite against oxidative stress. As the mannitol contents of conidia and mycelia from the different mutant genotypes were significantly different from that of the wild-type, the effects of *AbMpd* and *AbMdh* inactivation in *A. brassicicola* on conidia germination and initial mycelium growth in the presence of oxidative stressors were examined. Analysis of nephelometric growth curves revealed that under non-stress conditions (PDB medium), no significant phenotypic differences in conidia germination (based on the lag time parameter) or mycelium growth (based on the maximum growth rate parameter) were detected in any of the tested mutants as compared to the wild-type (data not shown). By contrast, Δ*abmdh* and Δ*abmpd* mutants, and to a lesser extent ΔΔ*abmpd-abmdh* mutants, were far more susceptible than the wild-type to 1 mM H_2_O_2_ treatments (Figure 5A). The Δ*abmpd* and Δ*abmpd-abmdh* mutants were also more susceptible to exposure to the superoxide-generating compound menadione than the wild-type and Δ*abmdh* genotypes (Figure 5B). As some brassicaceous defense metabolites have antifungal properties that might be at least partially linked to their capacity to generate oxidative stress (Sellam et al., 2007a), the susceptibility to allyl-ITC (Al-ITC), benzyl-ITC (Bz-ITC), and phenetyl-ITC (Ph-ITC) and brassinin were investigated (Figures 5C–F). A genotype susceptibility pattern similar to that obtained with menadione was observed in the presence of 5 mM Al-ITC, 5 mM Bz-ITC, 10 mM Ph-ITC and 200 μm brassinin. Note that after 24 h of exposure to ITC, the mannitol content of wild-type mycelia was 1.4-fold higher than in control and a 3-fold increased expression of *AbMpd* was recorded in cultures after 3 h of ITC treatment, while the *AbMdh* expression level remained unchanged (data not shown).

**Figure 6:**
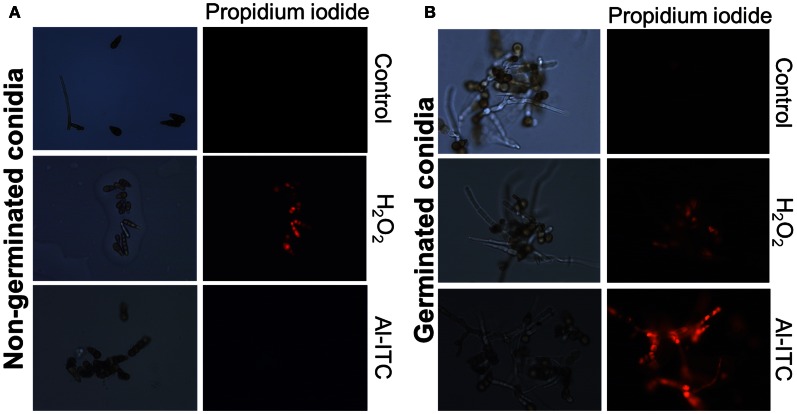
**Differential effect of H_2_O_2_ or Al-ITC on the viability of the *A. brassicicola* wild-type strain at different physiological stages.** Non-germinated conidia **(A)** and germinated conidia after 15 h of incubation **(B)** were treated with H_2_O_2_ (8 mM) or Al-ITC (5 mM) for 30 min or untreated (control) before observation. Suspensions were labeled with propidium iodide, which reveals dead cells, before fluorescence microscopy examination. This experiment was done twice with at least 100 spores or germlings and representative pictures are presented here.

Based on these susceptibility patterns, comparing the response of the four genotypes to H_2_O_2_ on one hand and to menadione or ITC on the other suggested different modes of action for these oxidants. This was further supported by comparing the effects on the cell viability of non-germinated and germinated conidia of low-time exposure to H_2_O_2_ and Al-ITC at their respective IC_50_ (Figure 6). On germinated conidia, treatment with Al-ITC resulted in PI-positive hyphae suggesting a loss of cell viability. No PI staining was observed in non-germinated conidia exposed to Al-ITC. Conversely, H_2_O_2_ caused cell death in non-germinated or germinated conidia.

**Figure 7:**
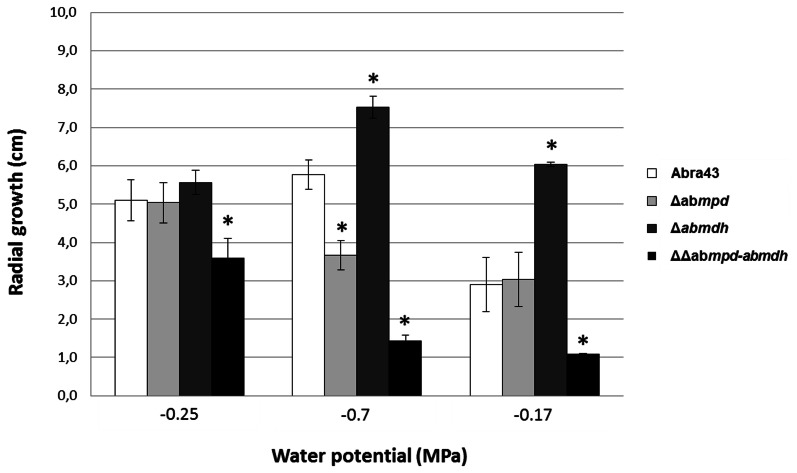
**Effect of low water potential treatments on the mycelium radial growth of the *A. brassicicola* wild-type strain and mannitol-metabolism mutants.** *In vitro* growth tests were carried out on PEG-infused plates (Verslues et al., 2006). Colony diameters (cm) were measured after 15 days of incubation at 24°C. Values are the means of three replicates. Error bars indicate standard deviations. Asterisks indicate a significant difference between the mutant and the parental isolate (Student test, *P* < 0.01).

Besides its potential role as antioxidant molecule, mannitol has been proposed to provide protection against drought stress (Dijksterhuis and de Vries, 2006). Media with different water potentials (from −0.25 MPa to −1.7 MPa) were prepared using a PEG-infused plate protocol (Verslues et al., 2006), inoculated with the different *A. brassicicola* genotypes and radial growth was recorded after 15 days of incubation. A shown in Figure 7, strains lacking functional MPD (Δ*abmpd* and ΔΔ*abmpd-abmdh*) were much more susceptible to low water potential treatments than the wild-type. Conversely, Δ*abmdh* mutants were more tolerant than the wild-type.

### Mannitol metabolism during plant colonization

**Figure 8:**
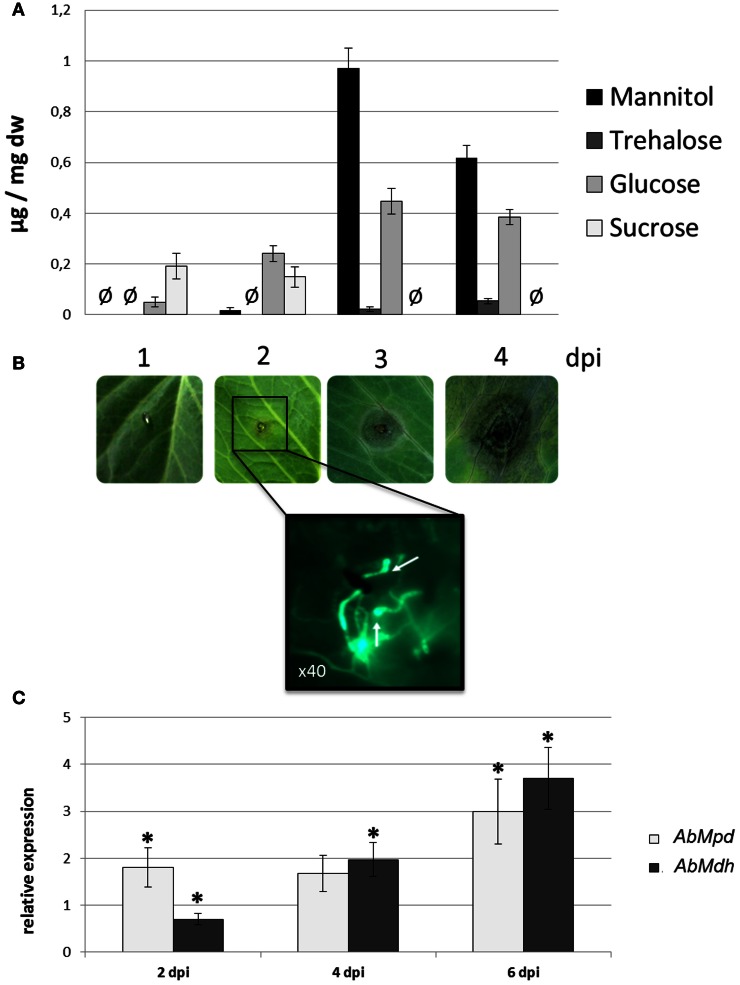
**Mannitol metabolism during plant colonization. (A)** Major soluble carbohydrate concentrations (expressed in μg/mg DF) measured by HPLC during the infection kinetics of the *A. brassicicola* wild-type strain on *Brassica oleracea*. Undetected sugars are represented by the symbol Ø. Three independent experiments were done. **(B)** Progression of symptoms on a *B. oleracea* leaf inoculated with the Abra43 strain and microscopic observations of the infection structures at 2 dpi. Inoculated plant tissue fragments were collected at 2 dpi and stained with solophenyl flavine for fluorescence microscopy observations. Appressoria-like structures are indicated by white arrows. **(C)** Quantitative RT-PCR results for the expression of *AbMpd* (white bars) and *AbMdh* (dark gray bars) during the infection kinetics of *A. brassicicola* wild-type strain on *B. oleracea*. For each gene, expression induction is represented as a ratio of its relative expression at 2, 4, and 6 dpi (studied gene transcript abundance/actin transcript abundance) in each inoculated sample to its relative expression in free-living fungal control cultures. The experiment was performed twice on biologically independent samples with three technical replicates. Error bars indicate standard deviations and asterisks indicate a relative expression significantly different from 1 (Student test, *P* < 0.01).

The HPLC profiles of the major soluble carbohydrates present over the course of *A. brassicicola* infection on *B. oleracea* leaves were established (Figure 8A). At 2 dpi, the wild-type strain produced characteristic appressoria-like structures at the tips of germ tubes in contact with the leaf epidermis (Figure 8B). Small necrotic symptoms were observed at 4 dpi, and they continued to expand into large typical necrotic areas surrounded by chlorotic halos at 6 dpi. At this infection stage, necrotic spots exhibited dense conidia formation on the surface. The HPLC analysis showed that, while the mannitol level remained below the detection limit in control plants, mannitol accumulated throughout infection and revealed a twenty-fold increase from 2 dpi to 4 dpi. A small decrease in mannitol was then observed at 6 dpi when sporulation was abundant. However, the mannitol level at this stage was still significantly higher than that detected at 2 dpi. Trehalose was only detected at low levels at 4 dpi and 6 dpi. Sucrose, which was the major carbohydrate in control non-inoculated plant samples, quickly decreased to undetectable levels at 4 dpi.

**Figure 9:**
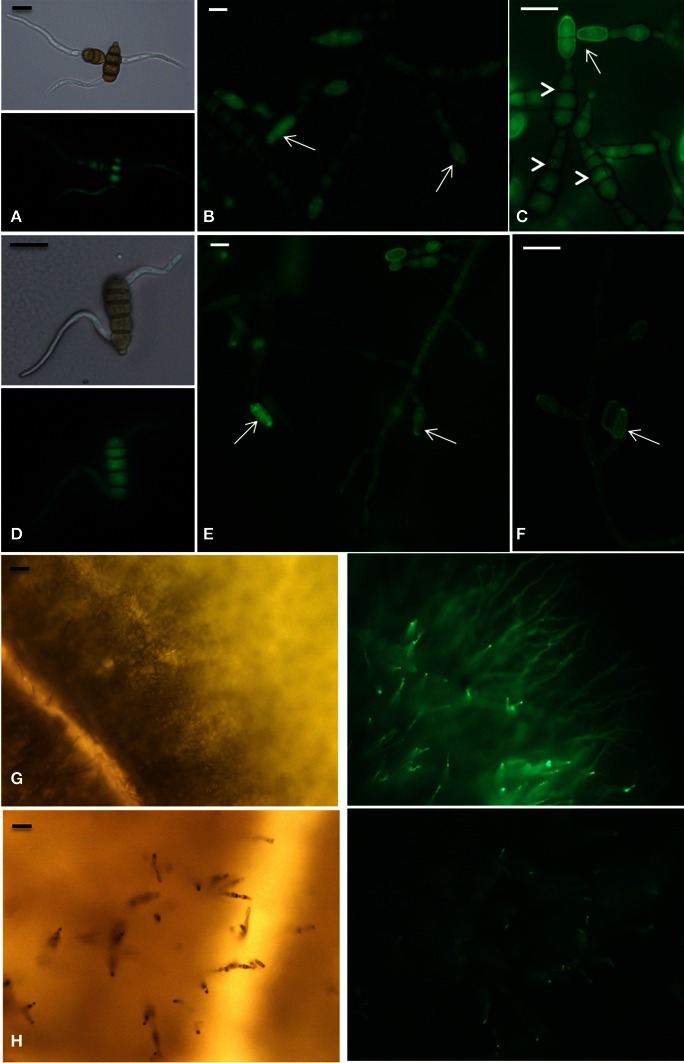
**Developmental phase-specific expression of AbMpd and AbMdh.** All panels are microscopy images of GFP expression in *A. brassicicola* strains expressing AbMdh (panels **A,B,C**, and **G**) and AbMpd (panels **D,E,F**, and **H**), under the control of their own promoter and fused at their carboxy-terminal end to SGFP. **(A)** and **(D)** Early germination stage 5 h after transfer to a solid PDA medium. Scale bars = 10 μm. (**B,C,E**, and **F**) Mycelia grown for 72 h on a PDA medium. At this stage, hyphae started to differentiate into conidiophores, leading to the production of young (arrow) and mature (arrowhead) conidia. Scale bars = 10 μm. **(G)** and **(H)** Fungal growth 6 days after inoculation of *B. oleracea* leaves. At this time, the fluorescence signal increased in aerial hyphae during the conidiation process. The right part corresponds to fluorescence microscopy and the left part to bright-field microscopy. Scale bars = 30 μm.

The expression of *AbMdh* and *AbMpd* during infection was also examined (Figure 8C). At 2 dpi, the expression level of *AbMpd* increased and remained higher than during *in vitro* growth over the time course of the experiment. Increased *AbMdh* expression was observed at a later stage and reached 3.5-fold its basal expression level at 6 dpi. These results are consistent with the metabolic profiling, which indicated a prevalence of mannitol production during plant infection. In order to follow the *in vitro* and *in planta* spatial expression patterns of both genes, strains expressing AbMpd and AbMdh, under the control of their own promoter and fused at their carboxy-terminal end to SGFP, were engineered. None of the transformants showed visible phenotypic changes discernible from the wild-type except for expression of green fluorescence (data not shown). In both strains, the fluorescence signal was detected in mature conidia and in the young germ tubes (Figure 9). In the *in vitro*-produced mature hyphae, the signal was still detectable but was much weaker in the AbMpd-GFP strain than in the AbMdh-GFP strain. Consistent with the *in planta* expression patterns, GFP fluorescence appeared to be stronger in the mutant during host plant infection. In AbMpd- and AbMdh–GFP fusion mutants, fluorescence intensity increased dramatically during the *in vitro* or *in planta* conidiation process, reaching a maximum in young conidia (Figure 9).

**Figure 10:**
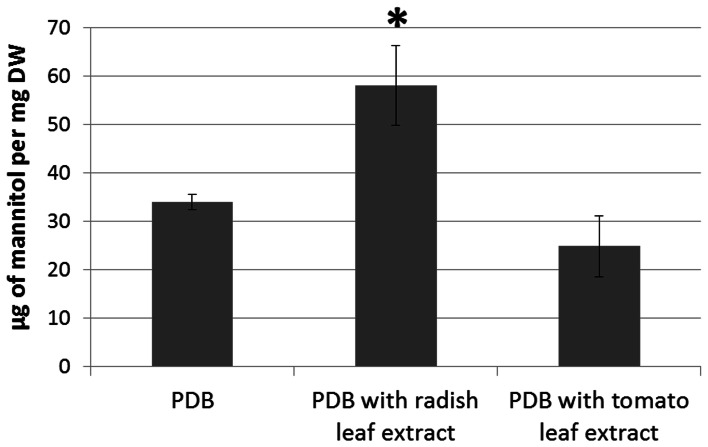
**HPLC assay of intracellular mannitol extracted from the mycelium of the wild-type genotype Abra43 grown in PDB supplemented or not with tomato or radish leaf extract (10% v/v) for 7 days.** Data represent the means of three independent experiments and error bars indicate standard deviations. Asterisks indicate a significant difference from the control culture conditions (PDB only) using the Student test (*P* < 0.01).

To test the hypothesis that host plants would elicit changes in fungal mannitol production, the *A. brassicicola* wild-type strain was cultured for 7 days in the presence and absence of leaf extracts from host (*B. oleracea*) or non-host (*Solanum lycopersicum*) plants. The amount of mannitol in the fungal mycelia was then determined by HPLC (Figure 10). Fungal growth was essentially unaffected by plant extracts (data not shown). *A. brassicicola* responded to the presence of host plant extracts by accumulating significantly higher levels of mannitol as compared to the amount detected in control culture or in the presence of non-host extract.

### Pathogenic behavior of replacement mutants on vegetative organs

**Figure 11:**
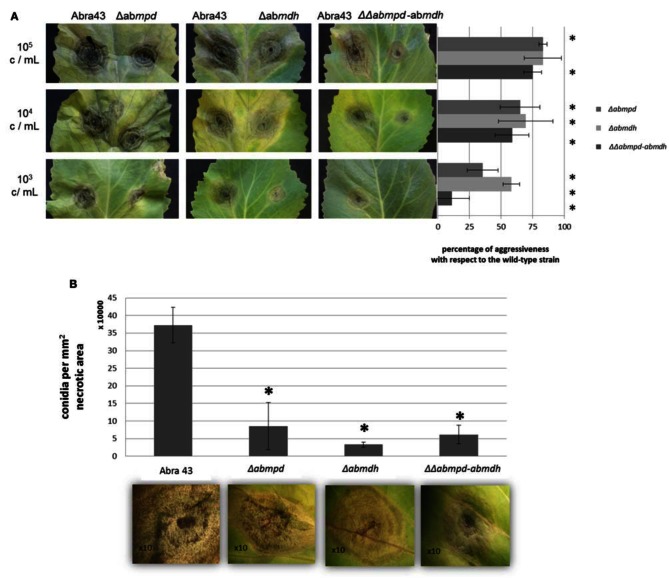
**Pathogenic behavior of replacement mutants on vegetative organs. (A)** Effects of targeted gene knockout in pathogenicity. *B. oleracea* leaves were inoculated with 5 μL drops of conidia suspension (10^5^, 10^4^, or 10^3^ conidia/mL in water). Mutants were inoculated on the right part of the central vein and compared on the same leaf with the parental strain (inoculated on the left part of the central vein). Percentage of aggressiveness with respect to the wild-type strain were calculated at 6 dpi by comparing the average lesion diameter obtained on five inoculated leaves. Asterisks indicate a significant difference with respect to the wild-type aggressiveness (100%) using the Student test (*P* < 0.01). **(B)** Effects of mutations in mannitol metabolism genes on the quantity of conidia produced *in planta*. The number of conidia is expressed per mm^2^ of necrotic *B. oleracea* leaf tissue at 6 dpi. For each genotype, five lesions were sampled and vortexed for 30 s in water containing Tween 20 (0.02%, v/v). The concentration of the resulting conidia suspensions was estimated microscopically using a haemocytometer. Error bars indicate standard deviations and asterisks indicate a significant difference with respect to the wild-type using the Student test (*P* < 0.01).

The accumulation of mannitol throughout infection of *B. oleracea* by *A. brassicicola* and the increased susceptibility of mannitol biosynthesis mutants to oxidative stress prompted us to comparatively evaluate the pathogenicity of the different fungal genotypes. The wild-type and Δ*abmdh*, Δ*abmpd*, and ΔΔ*abmpd-abmdh* mutants were all able to produce typical symptoms (Figure 11A). However, as judged from the lesion sizes at low inoculum charge, significant decreases in aggressiveness (up to 85% that of the wild-type at 10^3^ conidia per ml) were recorded for the ΔΔ*abmpd-abmdh* mutants and to lesser extent for the Δ*abmdh* and Δ*abmpd* mutants. Closer inspection of symptoms suggested that weak *in planta* sporulation occurred on necrosis obtained after inoculation with *AbMdh* deficient mutants (Figure 11B). This was confirmed by measuring the quantity of conidia produced per mm^2^ of necrotic tissue. All genotypes produced significantly fewer conidia *in planta* than the wild-type, with a 90% reduction for Δ*abmdh* mutants.

**Figure 12:**
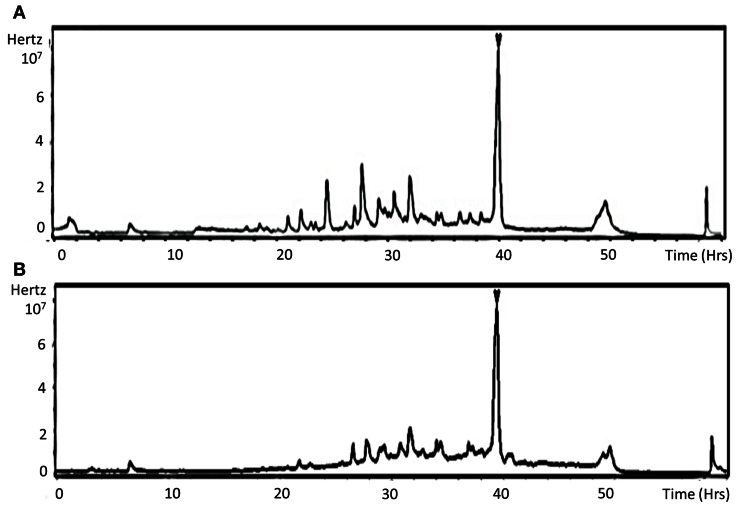
**Detection of the phytotoxin brassicicolin A in organic extracts from the culture broths of both the wild-type strain and the ΔΔ*abmpd-abmdh* mutant.** LC-MS total ion chromatograms (TICs) of Abra43 **(A)** and ΔΔ*abmpd-abmdh* **(B)** culture filtrate EtOAc extracts. Arrow indicates brassicicolin A. This experiment was done twice.

To check whether the lower aggressiveness of mutants could be correlated with *in planta* spore germination and/or plant tissue penetration defects, observation of solophenyl stained samples at 1 dpi was performed to quantify germinated conidia and appressoria-like structures at the plant surface. Although nearly 95% of conidia from all genotypes were germinated at this time, only 5 and 20% had differentiated infection structures in samples inoculated with the ΔΔ*abmpd-abmdh* and the Δ*abmpd* mutants, respectively, vs. 40% for samples inoculated with the wild-type or Δ*abmdh* mutants. Following host penetration, the fungus has to produce necrotic factors to progress within infected tissues. Brassicicolin A, a fungal metabolite considered as being a mannitol derivative, represents a potent necrotic toxin produced by *A. brassicicola* (Pedras et al., 2009). We hypothesized that the weak virulence in mutants lacking mannitol may also be explained by the absence of brassicicolin A synthesis. To test this hypothesis, ethyl acetate extracts of culture filtrates from submerged cultures of *A. brassicicola* wild-type and ΔΔ*abmpd-abmdh* strains were analysed by HPLC-UV-MS. The resulting total ion chromatograms (TIC) of both extracts revealed a major metabolite at *R*_t_ = 39 min (Figures 12A,B) exhibiting the quasimolecular ion [M-H]^−^ (*m/z* 683) of brassicicolin A (C_32_H_48_N_2_O_14_), whereas typical MS/MS fragments (1 V) were recorded at *m/z*, 565 (loss of two acetyl units) and 473 (loss of one α-hydroxyisovaleryl unit together with one α-isocyanoisovaleryl unit) (data not shown). The corresponding compound, which seems to accumulate in the same amounts in both strains, was purified through preparative TLC from the wild-type extract and analyzed (^1^H NMR, COSY and HMQC) in a capillary NMR probe (500 MHz). The resulting data (data not shown) were also in full agreement with former measurements obtained by Gloer et al. (1988) for this mixture of stereoisomers and confirmed that the isolated compound was brassicicolin A. We concluded that brassicicolin A was present in organic extracts from the culture broths of both the wild-type strain and the ΔΔ*abmpd-abmdh* mutant and that the attenuated virulence of mannitol-deficient mutants could not be linked to the loss of brassicicolin A production.

### Pathogenic behavior of replacement mutants on reproductive organs

**Figure 13:**
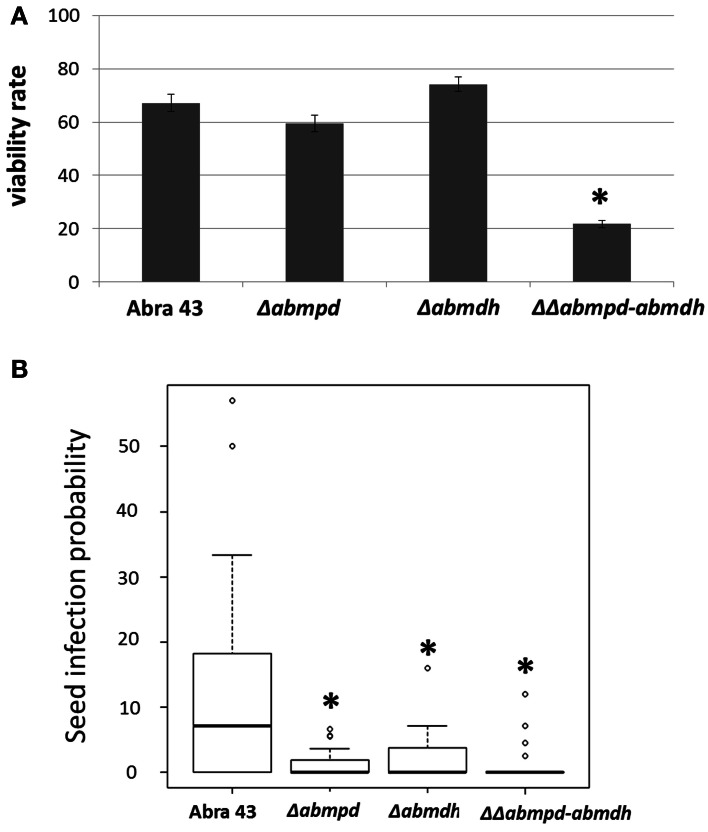
**Pathogenic behavior of replacement mutants on reproductive organs. (A)** Ability of the fungus to survive after storage on dry seeds. *B. oleracea* seeds were artificially inoculated by incubation in a conidia suspension of wild-type or mutant strains. The fungal viability rates were estimated by calculating the ratios between the nephelometric lag times obtained from seed batches before and after 6-months of storage. Ten seeds were analyzed for each fungal genotype and the experiment was repeated twice. Error bars indicate standard deviations. Asterisks indicate a significant difference between the mutant and the parental isolate (Student test, *P* < 0.01). **(B)** Influence of mannitol metabolism on the transmission capacity of *A. brassicicola* to *A. thaliana* seeds (L*er* ecotype). The seed infection probability was evaluated as described by Pochon et al. (2012). The five youngest siliques of at least five plants were inoculated with each fungal genotype and the experiment was repeated twice. Contaminated siliques were harvested 10 dpi. After dissection, seeds were incubated separately on PDA medium for 2 days. A seed was considered as contaminated when incubation resulted in typical *A. brassicicola* colony development. For each inoculated fungal genotype, the seed infection probability was evaluated from at least 1000 seeds. Asterisks indicate a significant difference between the mutant and the parental isolate (Student test, *P* < 0.01).

*A. brassicicola* is a seed-borne pathogen and has potential for long-term survival on dry seeds. As the differential abilities of the mutants to overcome a water potential stress have been observed, their capacity to survive on artificially contaminated seeds during storage was examined. After 6 months of storage, the percentage of viability was estimated using laser nephelometry growth curves on the basis of Δlag time values. As shown in Figure 13A, ΔΔ*abmpd-abmdh* mutant survival was significantly lower than that of other tested genotypes. Similarly, the ability of *A. brassicicola* to efficiently infect seeds has been correlated with their capacity to cope with severe stress conditions consecutive to gradual dehydration of maturating reproductive organs (Iacomi-Vasilescu et al., 2008). Using the model pathosystem recently described for investigating seed transmission in *Arabidopsis* plants (Pochon et al., 2012), the abilities of the mutants to transmit to seeds were compared with that of the wild-type. As shown in Figure 13B, the transmission capacity to *A. thaliana* seeds was significantly affected in Δ*abmpd* and Δ*abmdh* mutants and almost completely abolished in the ΔΔ*abmpd-abmdh* mutants.

## Discussion

The main goal of this study was to specify the roles of C6-polyol mannitol in the parasitic cycle of the fungal necrotroph *A. brassicicola*. We showed here that fungal mannitol over-accumulated in *B. oleracea* leaves during the interaction with *A. brassicicola*, as previously reported in *Arabidopsis* during *A. brassicicola* infection (Botanga et al., 2012) or for other necrotrophic (Jobic et al., 2007; Dulermo et al., 2009) and biotrophic (Voegele et al., 2005) interactions. In the latter type of interaction, accumulated mannitol might provide a means for the fungus to store carbohydrate in a form that is not accessible to the host and maintain a gradient of metabolites in favor of the pathogen (Voegele et al., 2005). Similarly to the finding of previous studies involving fungal necrotrophs (Jobic et al., 2007; Dulermo et al., 2009), sucrose dramatically decreased during host colonization by *A. brassicicola* and this plant specific carbohydrate was below the detection level at 6 dpi, suggesting that mannitol biosynthesis could be a general fungal strategy to rapidly mobilize plant sugars. As proposed by Solomon et al. (2006), accumulated mannitol may then provide the necessary substrates and energy required for conidiogenesis.

Mannitol dehydrogenase (MDH), which is mainly involved in mannitol mobilization, and mannitol-1-phosphate dehydrogenase (MPD) are two key enzymes of mannitol metabolism in fungi (Krahulec et al., 2011). The corresponding *A. brassicicola* encoding genes (*AbMpd* and *AbMdh*, respectively) have been identified and their expression monitored in *B. oleracea* infected leaves. The AbMpd- and AbMdh-GFP fusion analysis showed that the expression pattern is closely related to the conidiation and germination processes in *A. brassicicola*. GFP fluorescence indicated that both proteins exhibited a relatively similar expression pattern during host infection or *in vitro* growth: the maximum fluorescence intensity was reached in young conidia during the conidiation process. A high fluorescence intensity was also detected in mature conidia and into the young germ tubes, whereas the signal was weaker or undetectable in mature hyphae. Our results differ from previous observations in *Aspergillus niger* (Aguilar-Osorio et al., 2010), reporting a spatial differentiation of the expression of these two proteins: expression of MDHA and MDH activity were detected only in spores, while expression of MPDA and MPD activity were detected only in hyphae. These conflicting results may reflect functional differences among fungal enzymes involved in mannitol metabolism.

Gradual induction of *AbMdh* gene expression during infection was observed, as previously shown in the *U. fabae*-*V. faba* interaction (Voegele et al., 2005). The highest expression level measured was at 6 dpi, i.e., coinciding with decreased accumulation of mannitol, suggesting the mobilization of the polyol at this stage of infection may be a consequence of massive germination of newly formed conidia. It has indeed been shown in *B. cinerea* that mannitol rapidly degrades during spore germination and that such a catabolic process likely involves MDH activity (Dulermo et al., 2010). However, there is accumulating evidence that, contrary to what was postulated by Hult and Gatenbeck (1978), mannitol metabolism is not a cyclical process in fungi (Solomon et al., 2007; Dulermo et al., 2009). In line with this, MPD deficient *A. brassisicola* mutants still produced mannitol and thus AbMdh may also contribute to mannitol biosynthesis during infection. *Abmpd* expression was found to be induced at the earliest stages of the interaction and remained at a higher level than in control mycelia throughout the infection. According to the well-documented involvement of the MPD-dependent pathway in mannitol biosynthesis in fungi (Solomon et al., 2007), such *Abmpd* over-expression during tissue infection might be necessary for asexual *in planta* sporulation and pathogen propagation. In line with this hypothesis, *mpd*-deficient *A. brassicicola* mutants that failed to accumulate mannitol in hyphae were significantly compromised in their ability to develop conidia *in planta*. However, conidiation was also severely impaired in the *mdh*-deficient mutant that still accumulated high levels of intra-hyphal mannitol, thus questioning the direct relationship between mannitol content and asexual spore differentiation. Note that, as observed in *B. cinerea* (Dulermo et al., 2010), trehalose was detected by ^13^C NMR in all the studied genotypes except the *mdh*-deficient mutant. This sugar might therefore also be required to promote normal conidiation in *A. brassicicola*, in line with its demonstrated involvement in *S. nodorum* sporulation (Lowe et al., 2009). The role of stored mannitol in conidia germination also seems unclear. In conidia of all tested *A. brassicicola* mutants, a drastic decrease in mannitol to below the detectable level for the ΔΔ*abmpd-abmdh* strain was observed, but normal spore germination kinetics were recorded. Similar observations were reported for *A. niger* and *S. nodorum* (Ruijter et al., 2003; Solomon et al., 2005, 2006). In contrast, the capacity to accumulate mannitol in hyphae could be correlated with the ability to differentiate penetration (i.e., appressoria-like) structures as revealed by microscopic observation of plant tissue inoculated with *mdp*-deficient mutants in which no mannitol could be detected in young hyphae. This inability to efficiently produce penetration structures by strains lacking a functional MPD-dependent pathway was not observed in *A. alternata* (Velez et al., 2007), but probably at least partially explained the reduced aggressiveness of *A. brassicicola mpd*-deficient mutants.

Besides a possible role in this pathogenesis-related developmental process, mannitol may have other functions during plant-fungus interactions. One earlier reported function attributed to mannitol is protection against oxidative stress generated by the host plant defense system (Jennings et al., 1998). Oxidative burst is a general plant defense mechanism that occurs at a very early stage of the interaction (Parent et al., 2008). It is characterized by rapid accumulation of hydrogen peroxide in the extracellular space of plant tissues exposed to biotic stress (Wojtaszek, 1997). This ROS, besides its potential antimicrobial activity, might regulate induced cell death at the infection site, as shown in the *A. thaliana*–*A. brassicicola* pathosystem (Pogany et al., 2009). In our study, *A. brassicicola* was found to be relatively tolerant of physiologically compatible H_2_O_2_ concentrations. By contrast, mannitol metabolism mutants were all characterized by increased susceptibility to this ROS and lower mannitol content in conidia. As propidium iodide staining revealed that H_2_O_2_ induced cell death, even in non-germinated conidia, mannitol may accumulate in this organ and have a major protective role against oxidative stress generated by H_2_O_2_. The decreased aggressiveness on *B. oleracea* and the lower capacity to be transmitted to *Arabidopsis* seeds *via* siliques observed for all mutant genotypes could therefore be related to their increased susceptibility to oxidative burst during the early leaf or silique infection stage. At a later infection stage, i.e., during leaf or silique tissue colonization, *A. brassicicola* is also exposed to glucosinolate-derived ITC that induce intracellular ROS accumulation in fungal cells (Sellam et al., 2007a). The results of the present study strongly suggested that ITC cell toxicity is mainly exerted on germ-tubes and young hyphae rather than on conidia, thus confirming previously published observations (Sellam et al., 2007b). Interestingly, Δ*abmpd* and ΔΔ*abmpd-abmdh* strains that failed to accumulate mannitol in young hyphae were found to be hyper-susceptible to allyl-, benzyl- and phenetyl-ITC and also to menadione, a reference superoxide–generating molecule. Conversely, Δ*abmdh* strains that accumulated normal mannitol levels in hyphae were found to be as tolerant as the wild-type genotype. *In planta* assays were conducted on leaves of *Brassica olerace*a var. Bartolo and fruits of *A. thaliana* ecotype L*er* that both accumulated various glucosinolates. Thus, in addition to their increased susceptibility to extracellular ROS, the low aggressiveness and seed colonization capacity of MPD-deficient mutants may also be related to their failure to overcome the intracellular oxidative stress caused by ITC during leaf or silique colonization.

Besides the capacity to colonize fruit and seed tissues, efficient seed transmission required long-term survival of the seed-borne fungus on dry seeds teguments. Ruijter et al. (2003) proposed that the alcohol functions of mannitol could enable this polyol to mimic water molecules and participate in cell tolerance to water stress. In line with this, *A. brassicicola ΔΔabmpd-abmdh* mutants, i.e., with no detectable hyphal and conidial mannitol, were found to be highly susceptible to matricial stress generated by PEG and showed low viability rates after 1 month of storage under on dry seeds.

The altered mannitol metabolism observed in MPD- and MDH-deficient mutants may have other pleiotropic effects on *A. brassicicola* pathogenicity. During tissue invasion, necrotrophic pathogens such as *A. brassicicola*, synthesize phytotoxins that facilitate their spread within the infected organs (Thomma, 2003). Gloer et al. (1988) proposed that mannitol could be a precursor for the biosynthesis of brassicicolin A, which was later described as being the major host-selective phytotoxin produced by *A. brassicicola* (Pedras et al., 2009). Surprisingly, brassicicolin A was identified in organic extracts from the culture broths of both the wild-type strain and the ΔΔ*abmpd-abmdh* mutant. Our HPLC profile analysis revealed the presence of traces of mannitol in 1-week-old ΔΔ*abmpd-abmdh* cultures, suggesting that mannitol was still produced in minute amounts despite complete alteration of both mannitol biosynthesis pathways. As already suggested by Dulermo et al. (2010), this latter finding challenges the existence of one additional yet undescribed pathway which could participate in mannitol metabolism and, more specifically, in *A. brassicicola*, in brassicicolin A synthesis. We identified several candidate enzymes to perform mannitol synthesis through potentially other metabolic routes. First, a BlastP search pointed out an *A. brassicicola* sequence (AB1271) sharing homology with the NADP^+^-dependent D-mannitol dehydrogenase TbMDH, described in *Tuber borchii* and belonging to a distinct subfamily among the polyol dehydrogenase family (Ceccaroli et al., 2007). Dulermo et al. (2010) have already suggested its involvement in *B. cinerea* mannitol metabolism. Secondly, we also found potential homologs of mannose-6-phosphatereductase (M6PR), a key enzyme that is involved in mannitol biosynthesis in higher plants (Everard et al., 1997).

In conclusion, these results highlight the importance of mannitol metabolism with respect to the ability of *A. brassicicola* to efficiently accomplish key steps of its pathogen life cycle. At the earliest stages of plant infection, the differentiation of infection structures and fungal protection against extracellular ROS generated by oxidative burst were correlated with mannitol accumulation in hyphae and conidia, respectively. During tissue colonization, although rapid conversion of plant sugars into mannitol through hyphae invasion may not be directly linked to necrotic toxin production, the polyol probably participates in fungal protection against intracellular ITC-derived oxidative stress. It may also constitute a carbohydrate store that could be remobilized during late infection stages for the *in planta* conidiation necessary for efficient horizontal transmission of the pathogen. Lastly, mannitol could also be involved in vertical transmission (i.e., seed transmission) of the pathogen by conferring protection against dehydration and allowing long-term survival of the fungus on stored seeds.

### Conflict of interest statement

The authors declare that the research was conducted in the absence of any commercial or financial relationships that could be construed as a potential conflict of interest.
